# Alzheimer’s Disease and microRNA-132: A Widespread Pathological Factor and Potential Therapeutic Target

**DOI:** 10.3389/fnins.2021.687973

**Published:** 2021-05-24

**Authors:** Meng Zhang, Zhigang Bian

**Affiliations:** ^1^Department of Gerontology and Geriatrics, Shengjing Hospital of China Medical University, Shenyang, China; ^2^Department of Otolaryngology Head and Neck Surgery, Shengjing Hospital of China Medical University, Shenyang, China

**Keywords:** miR-132, Alzheimer’s disease, pathogenesis, biomarker, neuroprotection

## Abstract

Alzheimer’s disease (AD) is a common neurodegenerative disease in the elderly and is the most common type of dementia. AD is mostly gradual onset, and involves slow, progressive mental decline, accompanied by personality changes; the incidence of AD gradually increases with age. The etiology of AD is unknown, although it is currently believed to be related to abnormal deposition of amyloid β-protein (Aβ) in the brain, hyperphosphorylation of microtubule-associated protein tau, and the release of various cytokines, complements, activators and chemokines by cells. MicroRNAs (miRNAs) are a class of highly conserved non-coding RNAs that regulate gene expression at the post-transcriptional level, and manipulate the functions of intracellular proteins and physiological processes. Emerging studies have shown that miRNA plays an important role in regulating AD-related genes. MiR-132 is known as “NeurimmiR” due to its involvement in numerous neurophysiological and pathological processes. Accumulating pre-clinical results suggest that miR-132 may be involved in the progression of Aβ and tau pathology. Moreover, clinical studies have indicated that decreased circulating miR-132 levels could be used a potential diagnostic biomarker in AD. Here, we review the pathogenic role of miR-132 activity in AD, and the potential of targeting miR-132 for developing future therapeutic strategies.

## Introduction

Alzheimer’s disease is one of the most common neurodegenerative disease in the elderly; the main neuropathological hallmarks of AD are senile plaques (SP), neurofibrillary tangles (NFT), and loss of neurons (Iqbal et al., 2010; Bloom, 2014). AD is the most common type of senile dementia, and is characterized by recessive onset, slow progressive mental decline, and personality changes (Cerovic et al., 2019). The incidence of AD increases gradually with age (Herrera-Espejo et al., 2019). The etiology of AD is believed to involve abnormal deposition of amyloid β-protein (Aβ) in the brain (Chakravorty et al., 2019). The Aβ in AD is mainly produced by degradation of β-site amyloid precursor protein-cleaving enzyme 1 (BACE1). An increase in concentration and activity of BACE1 in the brain indicates over-expression of the *BACE1* gene (Mazdeh et al., 2018). Intracellular NFT are mainly composed of paired helical fibers (PHF). It has been found that hyperphosphorylation of microtubule-associated protein tau results in the formation of PHF, which form NFT, damaging the stability of the cytoskeleton and causing neurotoxicity (Sorrentino and Bonavita, 2007). A variety of cytokines, complements, their activators, and chemokines, such as COX-2 and complement factor H (CFH), are involved in the inflammatory response, leading to non-specific inflammatory cell infiltration, contributing to the pathogenesis of AD (Rivas-Arancibia et al., 2015; Williams et al., 2015). Currently, as the specific pathogenesis of AD is unknown in clinical practice, it is difficult to have a simple and effective method for early diagnosis (Zeng et al., 2019). The main therapeutic drugs for AD in clinical practice are cholinesterase inhibitors (CHEI) and selective antagonists of N-methyl-D-aspartic acid (NMDA) receptors (Reisberg et al., 2003; Wattmo and Wallin, 2017). However, due to the unclear pathogenesis of AD, the currently marketed therapeutic drugs can only delay the progression of the disease or slightly improve it, and there are no effective drugs that can reverse or prevent the progression of AD. Therefore, it is crucial to identify the molecular mechanism of AD pathogenesis and develop new and effective treatment methods.

MicroRNAs are a class of small non-coding single-stranded RNA molecules about 21–25 nt in length, which are usually involved in regulating transcription and expression of target genes at the post-mRNA transcription level. Single miRNAs can regulate up to 200 mRNAs, thus playing important roles in many key biological metabolic processes, such as cell growth, tissue differentiation, cell proliferation, embryonic development, and apoptosis (Dickson et al., 2013; Dong et al., 2015). Therefore, miRNA mutations, changes in miRNA synthesis, and dysfunction of miRNA and its target sites may provoke the development of diseases. In addition, some miRNAs are widely distributed in the central nervous system (CNS) and play important regulatory roles in neural development, differentiation and maturation. Deregulated expression of these miRNAs may lead to the development of various neurological diseases, including AD (Song and Lee, 2015; Maffioletti et al., 2019).

More specifically, significant reduction in miR-29a/b-1 expression was found in patients with AD, indicating an abnormal increase in BACE1 (Hébert et al., 2008). In addition, Peter et al., found that miR-107 levels in the temporal lobe were lower in patients with early AD, and there was a negative correlation between miR-107 and BACE1 mRNA levels (Nelson and Wang, 2010). In particular, miR-146a is upregulated in AD brains, causing upregulation of immune and inflammatory signals through IRAK1 and TRAF6, suggesting that miR-146a may be dysregulated and lead to the inflammatory response in AD (Ferguson-Chanowitz et al., 1990; Wang et al., 2012).

Importantly, one of the most abundant miRNAs in the brain is miR-132 (Klein et al., 2007) which was first discovered in mouse nerve tissue by Lagos-Quintana et al. (2002). Mature miR-132 is 22 bp in length and is processed from a precursor sequence of 66 bp. Human miR-132 is composed of two homologous miRNAs, hsa-miR-132-5p, and hsa-miR-132-3p. MiR-132 is evolutionary conserved and has the same sequence and structure in humans, rats, mice, apes and other species. MiR-132 has tissue specificity and is highly expressed in nerve-related tissues. MiR-132 is transcribed by the activity-dependent transcription factor cAMP-response element binding protein (CREB), and regulates axon, dendritic and spinal maturation in response to multiple signaling pathways (Klein et al., 2007; Magill et al., 2010). Sirtuin 1, encoded in humans by SIRT1, is best recognized as associated with longevity and aging (Jeong et al., 2011), miR-132 is shown to target SIRT1 (Strum et al., 2009). Deletion of miR-132 is shown to induce tau aggregation and impair mouse cognitive skills (Smith et al., 2015; Hansen et al., 2016). In mammals, the IGF-1 pathway affects the phenotype of aging (Kenyon et al., 1993). FOXO1 is one of the major components of IGF-1 axis (Pöllänen et al., 2010), and it is also a potential regulatory target gene of miR-132 (Budzinska et al., 2016). Pathologically, various studies have indicated that miR-132 is the most common downregulated miRNA in the postmortem AD brain, and is involved in the progression of Aβ and tau pathology (Lau et al., 2013; Patrick et al., 2017; Pichler et al., 2017).

**FIGURE 1:**
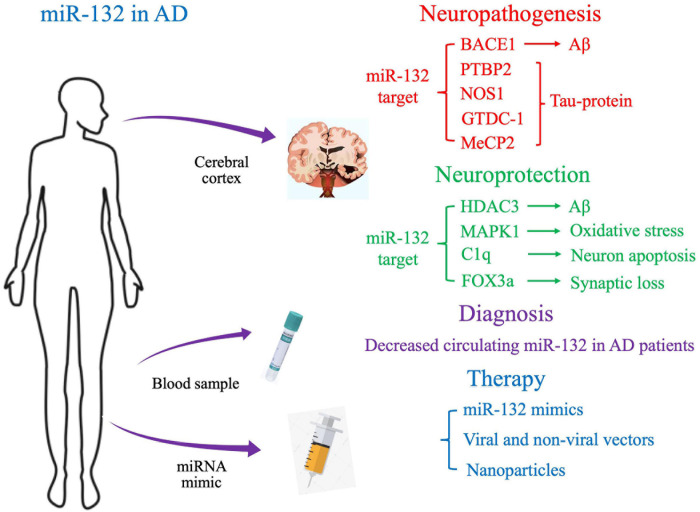
Proposed molecular targets and clinical application of miR-132 in Alzheimer’s disease.

In this review, we will discuss preclinical and clinical data on the novel role of miR-132 in AD pathophysiology, with the aim of gaining a deeper understanding of the molecular mechanisms of AD and developing novel therapeutic strategies (Figure 1).

## Neuropathogenesis and Neuroprotection of miR-132 in AD: Implications From *In Vivo* and *In Vitro* Studies

### Neuroprotection of miR-132 in AD

Downregulation of miR-132 has been shown to be involved in the pathogenesis of AD both *in vivo* and *in vitro* AD models, therefore, regulating miR-132 expression by different mechanisms may provide a new therapeutic strategy for AD treatment.

Soluble Aβ has been shown to decrease miR-132 expression and increase histone deacetylase (HDAC) levels in cultured primary neurons. In addition, Wei et al. (2020) indicated that there was a direct regulatory relationship between miR-132 and HDAC3. Upregulating miR-132 expression or downregulating HDAC3 levels could enhance hippocampal long-term potentiation and prevent hippocampal impairment by Aβ in AD mice. They also concluded that upregulated miR-132 expression or reduced HDAC3 signaling may counteract synaptotoxicity and lessen the progressive effects of Aβ accumulation during human brain aging (Wei et al., 2020).

Mitogen-activated protein kinases (MAPKs) are serine-threonine kinases that are highly expressed in the CNS. MAPK1 is involved in the increase of inflammation and apoptosis of neurons. Neuron apoptosis during AD is closely associated with the MAPK pathway (Li et al., 2019). In the study by Deng et al. (2020) miR-132 expression was significantly downregulated in an AD rat model, and MAPK1 expression was significantly upregulated; they suggested that miR-132 and MAPK1 had specific binding sites, and that miR-132 could inhibit MAPK1 expression. They concluded that miR-132 can inhibit hippocampal oxidative stress and iNOS expression by inhibiting MAPK1 expression to improve the cognitive function of AD rats (Deng et al., 2020).

Synaptic loss in the hippocampus and neocortex occurs throughout the pathological process of AD. In the early stage of AD (Zhang et al., 2014), Aβ-induced synaptic loss is the main cause, while in the late stage, accumulation of tau protein promotes synaptic degeneration, which is the key factor that leads to dementia (Shankar et al., 2008; Marciniak et al., 2017). Furthermore, C1q is a major protein in the classical complement cascade, and is highly expressed in synaptic regions of the CNS in AD patients (Bialas and Stevens, 2013). It has been reported that, in mouse models of AD, C1q levels were increased and associated with synaptic loss before apparent plaque deposition (Hong et al., 2016), and inhibition of C1q expression reduced the number of phagocytic microglia, and may be involved in early synaptic loss (Stevens et al., 2007). Xu et al. (2019) showed that miR-132 was significantly reduced in the brains of AD patients; they also found that upregulated miR-132 could increase expression levels of synaptic proteins in the temporal cortex of mice, and the same effect could also be obtained by suppressing C1q levels. Subsequently, they verified that C1q was genuinely regulated by miR-132 *in vivo*, and suggested that miR-132 could prevent synaptic loss by regulating C1q expression, which may provide new strategies for treating AD (Xu et al., 2019).

Samandari-Bahraseman et al. (2019) found that miR-132 decreased in the brain cells of AD patients when studying the mechanism of *Eremostachys labiosiformis* in the treatment of AD. This downregulation of miR-132 expression led to increased neuronal cell apoptosis. Accordingly, upregulating miR-132 expression could prevent neuronal apoptosis by directly regulating the expression of FOX3a, PTEN and P300 as the main components of the APK pathway (Samandari-Bahraseman et al., 2019).

Furthermore, El Fatimy et al. (2018) demonstrated that miR-132 exerted neuroprotective effects for AD through multiple signaling pathways; miR-132 could eliminate multiple forms of tau protein implicated in tauopathies. In addition, miR-132 could attenuate phospho-tau pathology and enhance long-term potentiation in P301S tau transgenic mice. Moreover, miR-132 protected neurons against Aβ and glutamate excitotoxicity; El Fatimy et al. (2018) indicated that miR-132 over-expression preserved cell body clusters and neurite integrity in PS19 neurons treated with Aβ. Collectively, these results demonstrated that miR-132 could regulate tau homeostasis, as well as protect neurons against Aβ deposition, suggesting that miR-132 supplementation could be therapeutically beneficial for treating AD (El Fatimy et al., 2018; Table 1).

**TABLE 1:** The role of miR-132 in Alzheimer’s disease: Evidence from *in vitro* and *in vivo* studies.

**Model**	**Expression**	**Target gene**	**Molecular mechanisms**	**Outcomes**	**References**
5XFAD mice	Downregulated	ITPKB	miR-132-ITPKB pathway	Increase BACE1 activity leading to more Aβ generation	Salta et al., 2016
Primary neuronal cells	Downregulated	PTBP2	Directly regulate PTBP2 expression	Affect endogenous 4R:3R-tau ratios	Smith et al., 2011
Primary neuronal cells	Downregulated	NOS1	Directly regulate NOS1 expression	Increase S-nitrosylation and induced tau phosphorylation	Wang et al., 2017
AD postmortem brain	Upregulated	GTDC-1	GTDC-1/CDK-5 signaling	Increase phosphorylation of Tau and apoptosis	Liu and Zhang, 2019
Mouse cortical neurons	Downregulated	MeCP2	Cycle of Tau-miR-132-MeCP2-Tau	Increase tau expression and phosphorylation	Xie et al., 2019
AD mice	Downregulated	Tau mRNA	Directly regulate Tau expression	Increased Tau expression, phosphorylation and aggregation	Smith et al., 2015
AD mice	Downregulated	HDAC3	Regulate HDAC3 signaling	Counteract synaptotoxicity and lessen the progressive effects of Aβ accumulation	Wei et al., 2020
AD rat	Downregulated	MAPK1	Regulate MAPK pathway	Inhibit hippocampal oxidative stress and iNOS expression	Deng et al., 2020
AD mice	Downregulated	C1q	Directly regulate C1q expression	Prevent synaptic loss in the temporal cortex	Xu et al., 2019
SH-SY5Y cells	Downregulated	FOX3a/PTEN/P300	Regulate APK pathway	Prevent neuronal apoptosis	Samandari-Bahraseman et al., 2019

Previous studies have found that several lncRNAs are specifically expressed in brain tissue, and are involved in many important neurological functions (Smalheiser, 2014; Barry et al., 2015; Lee et al., 2015; He et al., 2017; Yang et al., 2017). β-amyloid cleaving enzyme 1 antisense RNA (BACE1-AS) can promote tau phosphorylation, which is related to another important pathological sign of AD-NFTs. Ge et al. (2020) showed that miR-132-3p was a direct target of BACE1-AS, and was negatively regulated by BACE1-AS in neuronal cells. Depletion of BACE1-AS upregulated miR-132-3p expression and attenuated the neuronal damage induced by Aβ_25__–__35_, indicating that modulating the BACE1-AS/miR-132-3p axis could provide new insights into AD treatment (Ge et al., 2020). Wang et al. (2018) indicated that miR-132 was a target of XIST, and XIST could relieve Aβ_25__–__35_ induced toxicity, oxidative stress, and apoptosis in rat hippocampal neurons by upregulation of miR-132 expression, suggesting the potential of manipulating XIST in AD treatment.

In conclusion, these mechanisms may be applicable in the case of AD, and studying these potential miR-132 targets in AD may provide a promising area of research.

## Neuropathogenesis of miR-132 in AD

### The Effects of miR-132 on Aβ

It is believed that Aβ is the initiating factor of AD, and Aβ is hydrolyzed by β-amyloid precursor protein (APP); BACE1, as a lytic enzyme, plays an important role in the pathogenesis of AD. It has been shown that miR-132 is involved in regulating Aβ and BACE1 expression. Autopsy results have confirmed that BACE1 expression in the AD brain was upregulated at the protein level, but not at the mRNA level, further confirming that miRNA regulates gene expression after transcription rather than at the mRNA level (Ji et al., 2019).

Furthermore, Hernandez-Rapp et al. (2016) found that the amount of endogenous soluble Aβ_42_ in the hippocampus was significantly increased in 18-month-old triple transgenic AD (3xTg-AD) mice (with miR-132 knockout) compared to controls (without miR-132 knockout), suggesting that lack of miR-132 in mice can promote Aβ production, aggregation and deposition. In addition, regarding data from the Religious Orders Study (ROS), the researchers found that miR-132 levels were lower in mild cognitive impairment (MCI) and AD patients compared to healthy controls, and there was a negative correlation between miR-132 levels and memory deficits; miR-132 and insoluble Aβ_42_ levels were also significantly correlated (Hernandez-Rapp et al., 2016). To further verify the connection between miR-132 and Aβ, miR-132 mimics were established in Neuro2a and HEK293 APPSwe cell lines stably expressing (human) Aβ. In both cell lines, miR-132 significantly downregulated (soluble) human Aβ_40_ and Aβ_42_ levels (determined by ELISA), suggesting that miR-132 deficiency enhanced Aβ production (Hernandez-Rapp et al., 2016).

### The Effects of miR-132 on Tau Protein

Abnormal aggregation of tau protein is involved in the pathogenesis of AD, and the most common pathological changes in AD patients are tau protein phosphorylation, abnormal precipitation and aggregation of tau protein, resulting in the formation of NFTs (Congdon and Sigurdsson, 2018). A large number of specific miRNAs are reduced during AD development, which may be involved in the pathogenesis of AD by affecting the abnormal aggregation of tau protein (Fitzpatrick et al., 2017; Nisbet and Götz, 2018). MiR-132 directly targeted the neuronal splicing factor polypyrimidine tract-binding protein 2 (PTBP2); thus, miR-132 over-expression or knockdown of PTBP2 could affect endogenous 4R:3R-tau ratios in neuronal cells (Smith et al., 2011). Wang et al., suggested that miR-132 can directly regulate neuronal nitric oxide synthase (NOS1) expression through primate-specific binding sites. Inhibiting miR-132 expression in neural cells led to increased levels of NOS1 and triggered excessive production of nitric oxide, followed by abnormal S-nitrosylation of specific proteins related to tau pathology. This indicated that downregulation of miR-132 can disturb the balance of S-nitrosylation and induce tau phosphorylation in a NOS1-dependent manner, which may be involved in the pathogenesis of AD (Wang et al., 2017). Furthermore, Liu et al., showed that miR-132 expression was significantly higher in postmortem brain specimens of AD patients compared to normal controls. In addition, miR-132 could induce neuron apoptosis by affecting the expression of cell apoptosis-related factors, such as Bax and Bcl-2. Cyclic-dependent kinase-5 (CDK-5) is a critical kinase associated with tau phosphorylation. Over-expression of miR-132 was shown to promote CDK5 expression and accumulation of p35/p25. Glycosyltransferase-Like domain containing-1 (GTDC-1) was a direct target of miR-132, and over-expression of miR-132 could suppress GTDC-1 expression, inducing apoptosis of neuronal cells. In addition, GTDC-1 decreased Bax expression and increased Bcl-2 expression. Furthermore, GTDC-1 markedly inhibited tau phosphorylation. Therefore, miR-132 plays an important role in the pathogenesis of AD by regulating apoptosis and GTDC-1/CDK-5/tau phosphorylation signaling mechanisms (Liu and Zhang, 2019). Notably, in human tau-overexpressing neurons, methyl-CpG-binding protein 2 (MeCP2) levels were increased, while miR-132 was decreased, and miR-132 was found to negatively regulate MeCP2 expression both *in vitro* and *in vivo* (Xie et al., 2019). Xie et al., found that miR-132 deficiency led to increased tau expression, phosphorylation, and aggregation in mice. In addition, miR-132 regulated tau expression and phosphorylation, and thus contributed to tauopathy in AD by regulating MeCP2 levels, suggesting a vicious cycle of tau-miR-132-MeCP2-tau abnormalities in AD (Xie et al., 2019). Smith et al., found that miR-132 deficiency led to increased tau expression, phosphorylation and aggregation in mice, as well as demonstrating that miR-132 directly targeted tau mRNA to regulate its expression, by using reporter assays and cell-based studies. In addition, miR-132 mimics could partly restore memory function and tau metabolism in AD mice. Moreover, miR-132 levels were associated with insoluble tau and cognitive impairment in humans. MiR-132 could eliminate multiple forms of tau protein implicated in tauopathies, including the cleaved, phosphorylated and acetylated forms, and promote the extension and branching of neurites and reduce neuronal death. MiR-132 also directly regulated the tau modifiers acetyltransferase EP300, kinase GSK3β, RNA-binding protein Rbfox1, Caspases 3/7 and proteases Calpain 2; miR-132 could attenuate phospho-tau pathology and enhance long-term potentiation in P301S tau transgenic mice. These results support a role for miR-132 in the regulation of tau pathology in mice and humans, and may provide new strategies for therapeutic development (Smith et al., 2015).

## Biomarker Potential of Circulating miR-132 in AD

The etiology and pathogenesis of AD are not completely clear, and there are no effective means for early diagnosis and treatment (Cordell et al., 2013). As diagnostic biomarkers, both Aβ and tau protein are mainly expressed in cerebrospinal fluid (CSF), but collection of CSFs is invasive and inconvenient, making clinical development difficult. If reliable diagnostic biomarkers can be found in peripheral blood, it would be greatly significant for early screening, diagnosis and treatment of AD. MiRNAs are secreted extracellularly in the form of small vesicles or non-vesicles (such as peripheral blood, serum, plasma, saliva, and urine) and can bind to proteins or other compounds. For example, miRNAs in plasma can bind to high-density lipoproteins to form a stable structure and thus exist in peripheral blood (Noren Hooten et al., 2013; Turchinovich et al., 2013). Currently, miRNAs in the peripheral circulation are on the way to becoming important biomarkers for evaluating diseases (Schwarzenbach et al., 2014; Wang and Zhang, 2020).

Given the important role of miR-132 in the pathogenesis of AD, it is reasonable to suggest that it could serve as a potential biomarker for the diagnosis and progression of AD. A recent study of 24 AD patients and 45 controls showed that miR-132 levels in lymphoblastoid cell lines (LCLs) were dramatically decreased in AD patients compared with the controls. In addition, miR-132 expression levels were negatively correlated with Braak stage scores in AD olfactory bulb tissues (Hadar et al., 2018). Another study measured miR-132-3p levels in neurally derived plasma exosomes from 16 early stage AD patients, 16 MCI individuals and 31 healthy controls; Cha et al. (2019) indicated that miR-132-3p levels were lower in AD patients and MCI individuals compared to controls, thus demonstrating preferable sensitivity and specificity to diagnose AD. However, the results failed to separate AD patients from MCI individuals (Cha et al., 2019). Furthermore, miR-132 may also contribute to discriminate early cognitive dysfunction. Xie et al., performed a cross-sectional cohort study, which included 66 MCI patients and 76 healthy controls. They found that serum miR-132 levels were significantly increased in MCI patients compared with healthy controls. These results preliminarily suggested that circulating miR-132 was upregulated in MCI patients and could be potentially a biomarker for diagnosing MCI (Xie et al., 2015).

However, it is worth mentioning that, so far, there are relatively few studies that have investigated various circulating miRNA levels in AD patients, and all published data indicate that the overlap between detected miRNA targets is particularly limited. Moreover, the sample sizes of these studies were relatively limited, and the results were inconclusive. Most studies reported that miR-132 levels were downregulated in AD patients, and this abnormal expression appeared in the cerebral cortex and hippocampus of AD patients. In LCLs, miR-132 gradually decreased with age in patients with cognitive impairment. However, Xie et al., found serum miR-132 levels were significantly increased in MCI. Therefore, future studies are required to confirm these results, as well as the standardization of application methods, cohort verification from multiple centers, larger patient samples and ideal postmortem diagnosis of AD to obtain clinically meaningful results.

Therefore, miR-132 may be used as a diagnostic biomarker for AD, but in order to obtain clinically meaningful results, additional large sample studies are needed. In view of the importance of miR-132 in the pathogenesis of AD, even in the initial stage mentioned above, we could speculate that it may also be used as a preclinical biomarker in the pre-symptomatic stage, especially for high-risk groups.

## Therapeutic Potential of miR-132 in AD

Currently, AD treatment still has no efficient strategy to prevent the disease from progressing (Ayaz et al., 2017). Therefore, there is an urgent need to find new and effective therapeutic targets, especially regarding the early stages of the disease.

MicroRNAs have been shown to play an important role in biological processes such as cell differentiation, proliferation, apoptosis and other cell activities. In addition, a large number of experimental results have shown that miRNAs play a critical role in the development of neurons, muscles, and striatum (Follert et al., 2014). In recent years, studies have shown that miR-132, as an important regulator of neural development, is overexpressed in neural progenitor and mature neurons and can promote neural development (Chen D. et al., 2018; Jia et al., 2019). Currently, many miRNA-based treatment strategies have been developed, such as miRNA antisense technology and alternative therapies (Srivastava et al., 2016; Titze-de-Almeida et al., 2020). Antisense technology has been widely used to inhibit the expression of miRNAs. When there is over-expression of miRNAs in the body, which promotes disease development, antisense technology, namely miRNA inhibition therapy, can be used to block the expression of relevant proteins and thus inhibit miRNAs (Lima et al., 2018). Inhibitors of miRNA include anti-miRNA oligonucleotides (AMOs), which can be designed to complement and bind to miRNAs to prevent mRNA degradation (Lennox et al., 2017). It has been found that AMOs can enhance the selective hybridization of endogenous miRNA and achieve effective inhibition through chemical modification (Baumann and Winkler, 2014). Conversely, the development of miRNA mimics with the ability to bind to RNA-induced silencing complex and target mRNA as miRNA replacement therapy can further reduce mRNA expression levels (Lykhmus et al., 2016). These miRNAs mimics contain sequences consistent with those of mature endogenous miRNAs.

Gene therapy has received widespread attention as a way to treat certain diseases, such as cancer. miRNA therapy can be divided into two categories according to the expression status of the target miRNA: (1) miRNA inhibition therapy when miRNA is overexpressed; (2) miRNA replacement therapy when miRNA is inhibited (Roitbak, 2018). However, miRNA therapies still face a number of challenges, including achieving effective cell uptake and tissue-specific delivery, as well as issues of toxicity minimization and off-target effects. Therefore, miRNA-based therapies are highly dependent on utilizing vector-mediated delivery mechanisms. To improve the efficiency of miRNA delivery, viral and non-viral vectors have been developed (Wang et al., 2015; Labatut and Mattheolabakis, 2018). Virus-based delivery systems have high transduction efficiency and can effectively deliver genetic material to target cells. However, the disadvantages of viral vectors (e.g., inflammatory/immunogenicity response) limit their application in gene delivery and make it difficult to achieve large-scale manufacturing and quality control (Itaka and Kataoka, 2009; Chen Y. H. et al., 2018). Compared with viral vectors, non-viral delivery systems (polymer-based systems, liposomes and inorganic nanoparticles) are diverse and relatively safe, thus effectively avoiding the problems posed by viral vectors through reasonable design and appropriate modifications. Therefore, non-viral delivery systems can be widely used in clinical studies (Khalil et al., 2019; Zhang et al., 2019). Salta et al. (2016) showed downregulation of miR-132 in three distinct human AD patient cohorts, and showed that inositol 1,4,5-trisphosphate 3-kinase B (ITPKB) was a direct target gene of miR-132, which affected BACE1 activity, increasing Aβ expression in 5XFAD mice. This indicated the pathological relevance of the miR-132-ITPKB pathway in AD; considering the versatility of miRNAs as therapeutic drugs and the ever-increasing technical level, it was speculated that miR-132 mimics have potential applications in alleviating the progressive neurodegeneration of AD patients (Salta et al., 2016). In addition, Su et al. (2020) built wheat germ agglutinin (WGA)-nanoparticle-miR132 intranasally to treat AD mice, and found that nasal administration can enable the blood-brain barrier to be crossed, which increases drug bioavailability in the brain; moreover, synaptic protein expression levels were increased significantly after administration. Thus, proposal of the nasal delivery of WGA-nanoparticle-miR132 was an interesting novel therapeutic approach for AD treatment (Su et al., 2020).

However, as miR-132 may target various genes and be involved in multiple pathways in a cell-type-specific manner, its biological effects of miR-132-based therapy should be fully studied before they can be translated into clinical practice. Importantly, although emerging studies have investigated the potential role of various miRNAs in human disease, the most challenging part of the field is selecting the “correct” miRNA from a great number of potential candidates (Godlewski et al., 2019). Based on the above evidence, miR-132 is expected to become a target for AD therapy and thus merits further study.

## Conclusion

In conclusion, miR-132 is involved in the core pathophysiological mechanism of AD and may serve as a potential diagnostic biomarker. However, more studies are needed to successfully clarify its biological and clinical values. Although the effective delivery of miR-132 in the CNS has practical limitations, developing new strategies currently under study can pave the way for the clinical application of miR-132 for treating AD.

## Author Contributions

MZ and ZB drafted and revised the manuscript. ZB drafted and modified the figures. Both authors approved the final version of the manuscript and agreed to be accountable for all aspects of the work to ensure that questions related to the accuracy or integrity of any part of the work are appropriately investigated and resolved.

## Conflict of Interest

The authors declare that the research was conducted in the absence of any commercial or financial relationships that could be construed as a potential conflict of interest.
